# Activation of plant immunity by exposure to dinitrogen pentoxide gas generated from air using plasma technology

**DOI:** 10.1371/journal.pone.0269863

**Published:** 2022-06-24

**Authors:** Daiki Tsukidate, Keisuke Takashima, Shota Sasaki, Shuhei Miyashita, Toshiro Kaneko, Hideki Takahashi, Sugihiro Ando

**Affiliations:** 1 Graduate School of Agricultural Science, Tohoku University, Sendai, Miyagi, Japan; 2 Graduate School of Engineering, Tohoku University, Sendai, Miyagi, Japan; Hainan University, CHINA

## Abstract

Reactive nitrogen species (RNS) play an important role in plant immunity as signaling factors. We previously developed a plasma technology to partially convert air molecules into dinitrogen pentoxide (N_2_O_5_), an RNS whose physiological action is poorly understood. To reveal the function of N_2_O_5_ gas in plant immunity, *Arabidopsis thaliana* was exposed to plasma-generated N_2_O_5_ gas once (20 s) per day for 3 days, and inoculated with *Botrytis cinerea*, *Pseudomonas syringae* pv. *tomato* DC3000 (*Pst*), or cucumber mosaic virus strain yellow (CMV(Y)) at 24 h after the final N_2_O_5_ gas exposure. Lesion size with *B*. *cinerea* infection was significantly (*P* < 0.05) reduced by exposure to N_2_O_5_ gas. Propagation of CMV(Y) was suppressed in plants exposed to N_2_O_5_ gas compared with plants exposed to the air control. However, proliferation of *Pst* in the N_2_O_5_-gas-exposed plants was almost the same as in the air control plants. These results suggested that N_2_O_5_ gas exposure could control plant disease depending on the type of pathogen. Furthermore, changes in gene expression at 24 h after the final N_2_O_5_ gas exposure were analyzed by RNA-Seq. Based on the gene ontology analysis, jasmonic acid and ethylene signaling pathways were activated by exposure of *Arabidopsis* plants to N_2_O_5_ gas. A time course experiment with qRT-PCR revealed that the mRNA expression of the transcription factor genes, *WRKY25*, *WRKY26*, *WRKY33*, and genes for tryptophan metabolic enzymes, *CYP71A12*, *CYP71A13*, *PEN2*, and *PAD3*, was transiently induced by exposure to N_2_O_5_ gas once for 20 s peaking at 1–3 h post-exposure. However, the expression of *PDF1*.*2* was enhanced beginning from 6 h after exposure and its high expression was maintained until 24–48 h later. Thus, enhanced tryptophan metabolism leading to the synthesis of antimicrobial substances such as camalexin and antimicrobial peptides might have contributed to the N_2_O_5_-gas-induced disease resistance.

## Introduction

Developing agricultural systems that minimize environmental impacts remains a major challenge. Excessive use of chemical fertilizers and pesticides include the risks of contaminating the soil and harming the ecosystem [1]. The concept of Integrated Pest Management (IPM), which effectively combines a variety of control strategies rather than relying solely on chemical pesticides, has become widely accepted in the context of pest control [1]. These efforts can contribute to the achievement of the United Nation’s Sustainable Development Goals (SDGs). Therefore, it is desirable to develop further technologies to reduce the environmental impacts of agriculture and realize sustainable agricultural systems.

Plasma is a state of matter that can be observed as lightning or auroras in nature and is characterized by electrically charged energetic particles that can form highly reactive states such as radicals. Atmospheric pressure air plasma can be generated using air under atmospheric pressure with low electric power (<100 W) potentially supplied by renewable energy resources. Because of the low resource demands for its generation as well as its ability to generate biologically active reactive oxygen species (ROS) and reactive nitrogen species (RNS) [2], atmospheric pressure air plasma is attracting attention as a potentially sustainable technology in the fields of medicine and agriculture [3, 4].

Typical reactive species produced in atmospheric pressure air plasma include ozone (O_3_) as a ROS as well as nitric oxide (NO), nitrogen dioxide (NO_2_), and dinitrogen pentoxide (N_2_O_5_) as RNS [5–7]. It is well known that ROS and RNS are important signaling factors in the immune responses of plants. Plants produce ROS and RNS as a defense response when they perceive an infectious stimulus from a pathogen [8, 9]. The generated ROS and RNS function as signaling molecules that contribute to the activation of plant immunity [8, 10]. The functional ROS produced by plant cells include superoxide anion (O_2_^−^), hydroxyl radical (OH), hydrogen peroxide (H_2_O_2_), and singlet oxygen (^1^O_2_), among others. O_2_^−^ and H_2_O_2_ have been of particular interest in studies of the mechanisms of plant disease defense [11]. Also, RNS such as NO are produced during plant immune responses [12, 13]. Plant hormones such as salicylic acid (SA), jasmonic acid (JA), and ethylene (ET) are generally known to play important roles in the regulation of plant immunity [14], and many studies have reported that ROS and RNS signals are linked to the activities of these plant hormone signals [15].

Some reports indicate that exogenous application of RNS enhances plant disease resistance. Treatment with NO-releasing compounds appears to suppress tobacco mosaic virus infection in tobacco and rice black-streaked dwarf virus infection in rice through salicylic acid-mediated resistance [16, 17]. Furthermore, exposure of *Arabidopsis* to NO_2_ gas enhances basal resistance to *Botrytis cinerea* and *Pseudomonas syringae* by activating SA and JA signaling [18]. Exposure to high concentrations of NO_2_ gas, but not NO gas, induces cell death in *A*. *thaliana* [19]. When used to treat plants, NO_2_ gas is known to convert to nitrate (NO_3_^−^) and nitrite (NO_2_^−^). Thus, NO_3_^−^, NO, and H_2_O_2_ might act in a coordinated manner to regulate NO_2_-induced cell death [19]. Other reports show that NO and NO_2_ induce protein *S*-nitrosylation and tyrosine nitration, thereby regulating protein functions such as plant immunity and cell death [9, 20, 21].

Dinitrogen pentoxide is an active nitrogen species whose physiological effects are poorly understood due to difficulties with its synthesis and storage. We have developed a device that uses plasma technology to generate an active species gas containing a high concentration of N_2_O_5_ [6]. The gas generated using the plasma technology, which we call “N_2_O_5_ gas” in this study, contains a certain amount of O_3_ and NO_2_ in addition to its high concentration of N_2_O_5_ due to the nature of the matter. However, because the N_2_O_5_ gas we generate using this system has unique properties that cannot be created using conventional technology at present, it is meaningful to gain insight into its physiological effects. Although N_2_O_5_ readily dissolves in water and results in nitric acid (HNO_3_) *via* reactive intermediates [22], the effects on plants of such N_2_O_5_-induced chemical reactions are unknown. Moreover, there is currently no knowledge of plant responses after exposure to N_2_O_5_ gas. In the present study, we exposed *Arabidopsis* to plasma-generated N_2_O_5_ gas and analyzed its effects on disease resistance and post-exposure changes in gene expression after the exposure in order to elucidate the effects of N_2_O_5_ gas on plant immune responses.

## Materials and methods

### Dinitrogen pentoxide gas generation from air

Dinitrogen pentoxide gas, which is highly reactive, not storable under ambient conditions, and not typically available in the gas market, was prepared by an atmospheric pressure plasma device, developed recently in our previous work as shown in S1A Fig [6]. This device, which has electric controls designed for plant exposure experiments, can produce a continuous supply of N_2_O_5_ gas from only compressed air via a chemical reaction chain involving N_2_O_5_, NO_2_, and O_3_. Importantly, to prevent contamination by any chemicals, no chemical compounds were used for the generation N_2_O_5_ gas by the given plasma device. Furthermore, exactly the same conditions without reactive species were created by turning off the generation of air plasmas by the plasma device. Due to the unavoidable generation and decomposition reaction chains involving N_2_O_5_ under physiological conditions, N_2_O_5_ selectivity is limited up to 10 at a density of approximately 240 ppm, which allows simultaneously high density and high selectivity at room temperature. The minor gas components of O_3_, NO_2_, N_2_O, and HNO_3_ that we measured that were unavoidably present in the N_2_O_5_ gas are summarized in S1C Fig [6]. This N_2_O_5_ gas mixture synthesized from air is henceforth described in this paper simply as N_2_O_5_ gas.

### Plant cultivation and N_2_O_5_ gas exposure of plants

Wild-type plants of *Arabidopsis thaliana* ecotype Columbia (Col-0) and the mutants *coi1-1*, *ein2-1*, and *npr1-1* in the same background were sown on soilless mix (Metro-Mix 350, San Gro, Canada) and grown for 2 weeks. Each seedling was then transferred to a new pot for further cultivation for 3 weeks in a growth chamber under short day conditions (light 10 h/dark 14 h) at 23°C. Homozygous *coi1-1* plants were screened using a dCAPs marker before transplanting [23]. The N_2_O_5_ gas generated by the transportable plasma device was used for exposing plants to N_2_O_5_ gas [6]. The plants were incubated for 30 min under a clear cover prior to exposure to N_2_O_5_ gas to ensure uniform humidity conditions. Dry air containing N_2_O_5_ at a density of approximately 240 ppm was emitted at 2 L/min from the outlet of a polytetrafluoroethylene (PTFE) tube with a 4-mm inner diameter. Each *A*. *thaliana* pot was placed 5 cm downstream from the N_2_O_5_ gas outlet tube for 20 s once per day with an outer plastic shroud to prevent room air flow disturbances from causing unexpected processes as shown in S1B Fig.

### *Botrytis cinerea* inoculation

*Botrytis cinerea* isolated from *Brassica* species (MAFF 237695) was provided from NARO GeneBank [24]. *Botrytis Cinerea* was cultured on potato dextrose agar (Difco, Detroit, MI) medium at 23°C for 3 days under dark conditions, and then plates were transferred to incubate under continuous black light (FL10BLB; Toshiba Corp., Tokyo, Japan) for 3 days to induce conidia formation. Conidia were suspended in potato dextrose broth (Difco) using a paint brush and then filtered through four layers of gauze. Conidial suspensions were centrifuged at 400 × *g* for 5 min and the supernatant was removed. The collected conidia were resuspended in 1/8 diluted potato dextrose broth to a concentration of 2 × 10^5^ conidia/mL. Five-week-old plants were exposed to N_2_O_5_ gas once per day for 3 days, and the plants were inoculated with *B*. *cinerea* at 24 h after the final N_2_O_5_ gas exposure. A separate set of control plants were sprayed with 200 μM methyl jasmonate (MeJA) and incubated for one day under a clear cover for comparison with the effect of the N_2_O_5_ gas. The conidial suspension (5 μL) was spotted onto a fully expanded leaf and incubated for 2 days while maintaining high humidity. The area of each lesion (mm^2^) was measured to evaluate disease severity. Trypan blue staining was performed to detect dead cells, as previously described [25]. Statistical analyses were performed using Student’s *t*-test or the Tukey–Kramer test, depending on the number of experimental groups. Each experiment was performed at least twice and similar results were obtained each time.

### Inoculation with *Pseudomonas syringae* pv. *tomato* DC3000

Five-week-old plants were exposed to N_2_O_5_ gas once per day for 3 days, and then *Pseudomonas syringae* pv. *tomato* DC3000 (*Pst*) was inoculated at 24 h after the final gas exposure. King’s B liquid medium supplemented with 50 μg/mL of rifampicin was used for *Pst* culture at 25°C for 1 day and the bacterial concentration was adjusted with 10 mM MgCl_2_ solution to an OD_600_ value of 0.002. The bacterial suspension was infiltrated into the intercellular spaces of three fully expanded leaves per plant using a syringe. Mock treatment was performed by infiltration with 10 mM MgCl_2_ in the absence of bacteria. The inoculated leaves were sampled and ground using a pestle in a tenfold volume of sterile water at 2 days after inoculation. Successive dilutions of the ground leaf tissue were spread onto King’s B solid medium supplemented with rifampicin (50 μg/mL), incubated at 25°C for 2 days, and the number of *Pst* colonies formed was counted. In addition, bacterial biomass was assessed by calculating the ratio of bacterial DNA to plant DNA using qPCR. Inoculated leaves were collected at 0, 1, 2 and 3 days after infection and total DNA was extracted using an ISOPLANT II kit (Nippon Gene Co., Tokyo, Japan) according to the manufacturer’s protocols. To quantify plant DNA and *Pst* DNA, *RHIP1* and *OprF* sequences [26], respectively, were amplified by qPCR using TB Green^®^ Premix Ex Taq™ II (Takara Bio Inc., Shiga, Japan) on a 7300 Real-Time PCR System (Applied Biosystems, Foster City, CA) (S1 Table). Student’s *t*-test was performed for statistical analysis and no significant differences were found. Each experiment was performed three times with similar results.

### Inoculation with cucumber mosaic virus

Cucumber mosaic virus strain yellow (CMV(Y)) was inoculated and propagated on *Nicotiana benthamiana* plants and purified as described [27]. Five-week-old *Arabidopsis* plants were exposed to N_2_O_5_ gas once per day for 3 days, and were inoculated with CMV(Y) at 24 h after the final N_2_O_5_ gas exposure. Three leaves of the *Arabidopsis* plants were inoculated with CMV(Y) as described [28]. Briefly, mechanical inoculation of the virus was carried out onto leaves sprinkled with carborundum by rubbing the surface lightly with a cotton swab soaked in virus solution. Air exposure was used as control and was also subjected to CMV(Y) inoculation or mock treatment (water). An enzyme-linked immunosorbent assay (ELISA) was performed as described previously to quantify CMV(Y) multiplication [29]. A rabbit antibody against the CMV(Y) coat protein (CP) and alkaline phosphatase-conjugated anti-rabbit IgG (Fc) (Promega, Madison, WI) were used as the primary and secondary antibodies, respectively. The compound *p*-nitrophenyl phosphate (1 mg/mL) in AP9.5 buffer (10 mM Tris-HCl [pH 9.5], 100 mM NaCl, 5 mM MgCl_2_) was used as the substrate for alkaline phosphatase. The absorbance of the resulting phenolate solution was measured at 405 nm. The amount of CP in 0.025 mg total protein was calculated as average absorbance ± standard deviation. Student’s *t*-test was performed for statistical analysis. Each experiment was performed at least twice with similar results.

### Transcriptome analysis by RNA-seq

Five-week-old plants were exposed to N_2_O_5_ gas for 20 s once per day for 3 days, and fully expanded leaves were sampled at 24 h after the final exposure to N_2_O_5_ gas (hereafter, N_2_O_5_-gas-exposed). Plants exposed to air served as the control experiment (hereafter, Air-control). Total RNA was extracted from leaves using the TRIzol method [30]. Macrogen Japan on the Illumina platform was used for RNA-seq analysis in order to obtain 151-bp paired-end sequences. Fifty-one M reads were obtained for the Air-control, and 46 M reads for the N_2_O_5_-gas-exposed sample were obtained. Differentially expressed gene analysis was performed between the Air-control and N_2_O_5_-gas-exposed samples. Fold changes (fc) in transcript abundances were calculated using exactTest in the edgeR package [31] for each sequence pair comparison. Significant results are indicated with |fc|≥2 at an exactTest raw *p*-value <0.05. Functional category enrichment was defined by implementing the Gene Ontology (GO) tool online (http://geneontology.org/). The data for RNA-Seq have been deposited in the DDBJ Sequence Read Archive (DRA) (https://www.ddbj.nig.ac.jp/dra/index-e.html) and are accessible through DRR Run accession numbers: DRR345814 and DRR345815.

### Gene expression analysis by qRT-PCR

Total RNA was extracted from individual seedlings using the TRIzol method [30]. Reverse transcription and subsequent PCR were performed using PrimeScript™ RT Reagent Kit with gDNA Eraser (Takara Bio Inc.). The relative abundances of mRNA transcripts for each gene of interest were determined by qRT-PCR using TB Green^®^ Premix Ex Taq™ II (Takara Bio Inc.) and a 7300 Real-Time PCR system (Applied Biosystems). Transcript abundance was calculated and represented as fold difference relative to the abundance of *ACTIN2* transcripts. The average and standard deviation of values of three independent seedlings were then calculated. Student’s *t*-test, Dunnett’s test, or Tukey–Kramer test were performed for statistical analysis depending on the number and character of experimental groups. Each experiment was performed at least twice with similar results. Primers used in this study are listed in S1 Table.

### Statistical analysis

All data were subjected to analysis of variance and various post-hoc tests using R version 3.6.3 (R Foundation for Statistical Computing, Vienna, Austria).

## Results

### Determination of N_2_O_5_ gas exposure conditions for *Arabidopsis thaliana*

**Fig 1:**
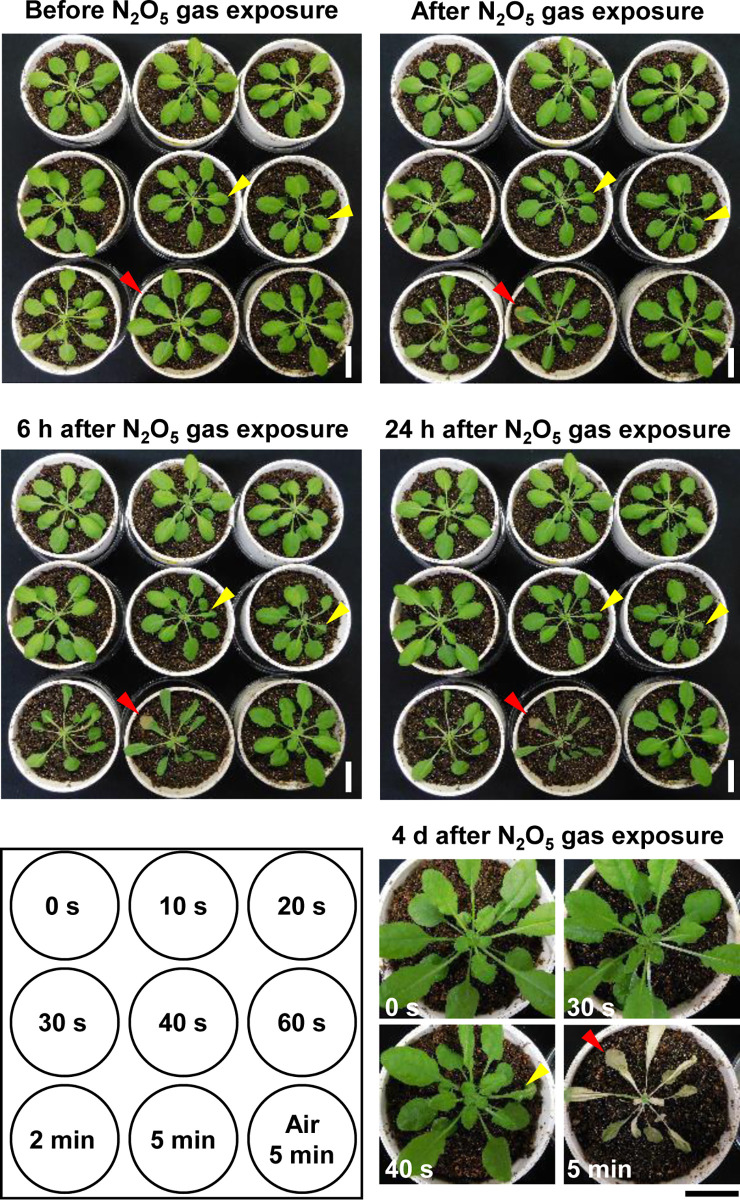
Observation of plant damage after exposure of *Arabidopsis* plants to N_2_O_5_ gas. The N_2_O_5_ gas exposure time for each plant is shown in the lower left corner. Observations were made before, immediately after, 6 h after, 24 h after, and 4 days after N_2_O_5_ gas exposure. Magnified images at 4 days after some of the N_2_O_5_ gas exposure times (0 s, 30 s, 40 s, and 5 min) are shown in the lower right corner. Red arrowheads indicate a leaf that turned brown immediately after the N_2_O_5_ gas exposure. Yellow arrowheads indicated the location of leaves that were damaged by the N_2_O_5_ gas. Scale bar is 2 cm.

To determine the N_2_O_5_ gas exposure conditions that would not be harmful for plant growth, *A*. *thaliana* plants were exposed to N_2_O_5_ gas by placing them 5 cm downstream from the gas outlet tube for 0 s, 10 s, 20 s, 30 s, 40 s, 60 s, 2 min, or 5 min. Immediately after the N_2_O_5_ gas exposure for 300 s, leaves turned brown and died by 24 h after exposure (Fig 1). Clear injury was observed with N_2_O_5_ gas exposure of 40 s at 6 h post-exposure (Fig 1). However, no apparent change in leaves was observed with up to 30 s of N_2_O_5_ gas exposure even at 4 days after exposure (Fig 1). We have also confirmed that N_2_O_5_ gas exposure for 20 s repeated once per day for 3 days did not cause apparent injury to plants. Therefore, exposure to N_2_O_5_ gas for 20 s was chosen as the experimental treatment for the following analyses.

### Effects of the N_2_O_5_ gas exposure on disease resistance

**Fig 2:**
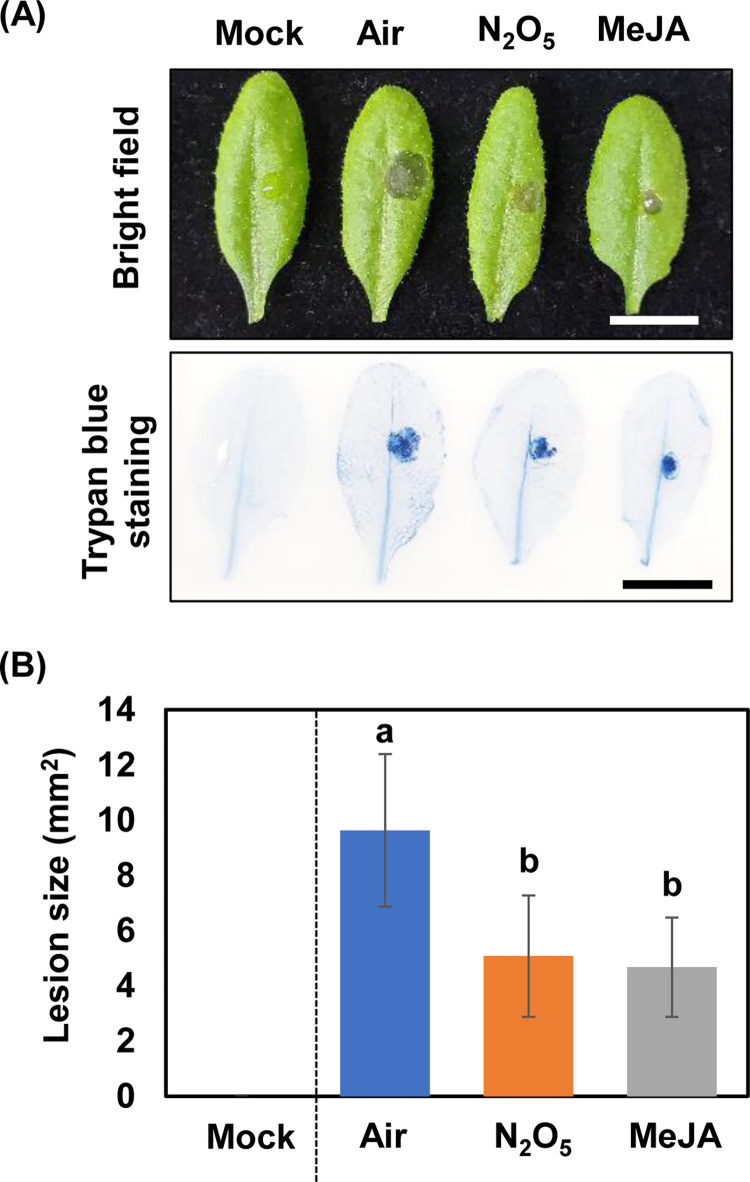
Induction of *Botrytis cinerea* resistance in *Arabidopsis* plants by N_2_O_5_ gas exposure. (A) *Arabidopsis* plants exposed to N_2_O_5_ gas were inoculated with *B*. *cinerea*. Scale bar is 1 cm. (B) Areas of the lesions at 2 days after *B*. *cinerea* inoculation were measured and the mean (± standard deviation) is shown. Different letters indicate significant differences according to the Tukey–Kramer test (n = 11–12, *P* < 0.05).

Air-exposed and N_2_O_5_-gas-exposed plants were inoculated with *B*. *cinerea*. The lesions were smaller on the N_2_O_5_-gas-exposed plants than on the control plants at 2 days post-inoculation (Fig 2). Trypan blue staining also confirmed that the area of dead tissue caused by *B*. *cinerea* infection was smaller in the N_2_O_5_-gas-exposed plants than in the control plants (Fig 2A). Measurements of lesion diameter confirmed that N_2_O_5_-gas-exposed plants reduced lesion size to approximately 53% of the control. Furthermore, the size of lesions on the N_2_O_5_-gas-exposed plants was similar to that on the MeJA-treated plants (Fig 2A). Therefore, N_2_O_5_ gas exposure appears to enhance the *B*. *cinerea* resistance of *A*. *thaliana* to the same extent as does MeJA.

**Fig 3:**
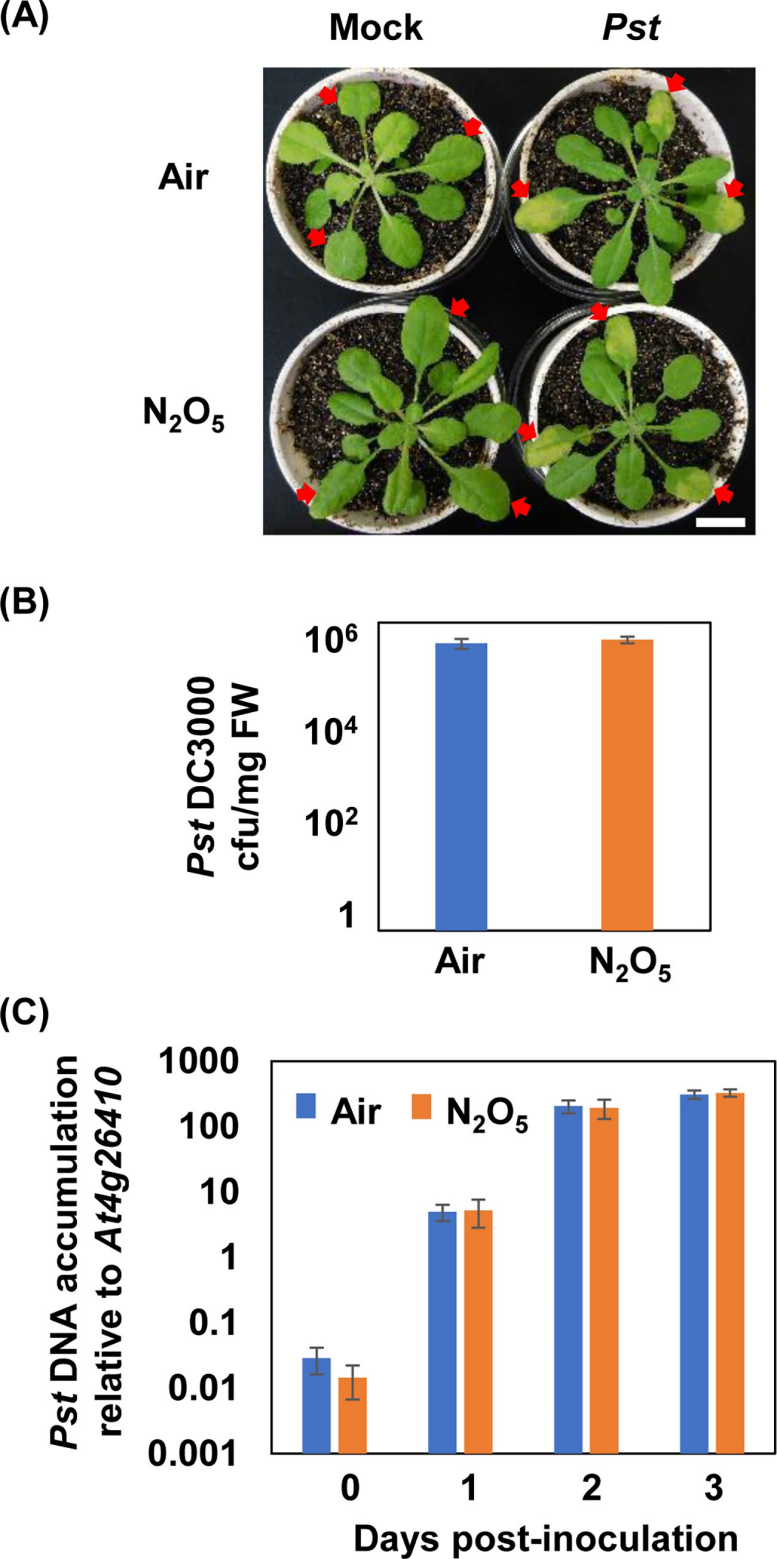
Response of N_2_O_5_-gas-exposed *Arabidopsis* plants to infection with *Pseudomonas syringae* pv. *tomato* DC3000. (A) Lesion formation was observed at 3 days after *Pst* inoculation. Red arrows indicate inoculated leaves. Scale bar indicates 1 cm. (B) Inoculated leaves were collected at 2 days after infection and the bacterial titer (cfu/mg fresh weight of leaf material) was determine by the colony counting method. The graph shows means (± standard deviation) of the results from three independent plants. No significant differences between the treatments were detected (Student’s *t*-test, *P* < 0.05). (C) Plant biomass and bacterial biomass were calculated by qPCR and the ratio of bacterial DNA per plant DNA was shown. There were no significant differences between treatments at each time point (Student’s *t*-test, n = 3, *P* < 0.05).

Next, separate sets of N_2_O_5_-gas-exposed and air-exposed plants were inoculated with *Pst*. Chlorosis was clearly observed on the inoculated leaves of both N_2_O_5_-gas-exposed and air-exposed plants at 3 days post-inoculation (Fig 3A). Analysis of bacterial growth by culture methods showed no effect of the N_2_O_5_ gas exposure after 2 days of inoculation (Fig 3B). Furthermore, the results of qPCR analysis of bacterial biomass over time showed no effect of exposure to N_2_O_5_ gas on bacterial growth (Fig 3C). It was observed that the growth of *Pst* was intense up to 2 days post-inoculation and reached saturation by 3 days post-inoculation in both treatments. These results indicate that *Pst* resistance is not enhanced by the N_2_O_5_ gas exposure.

**Fig 4:**
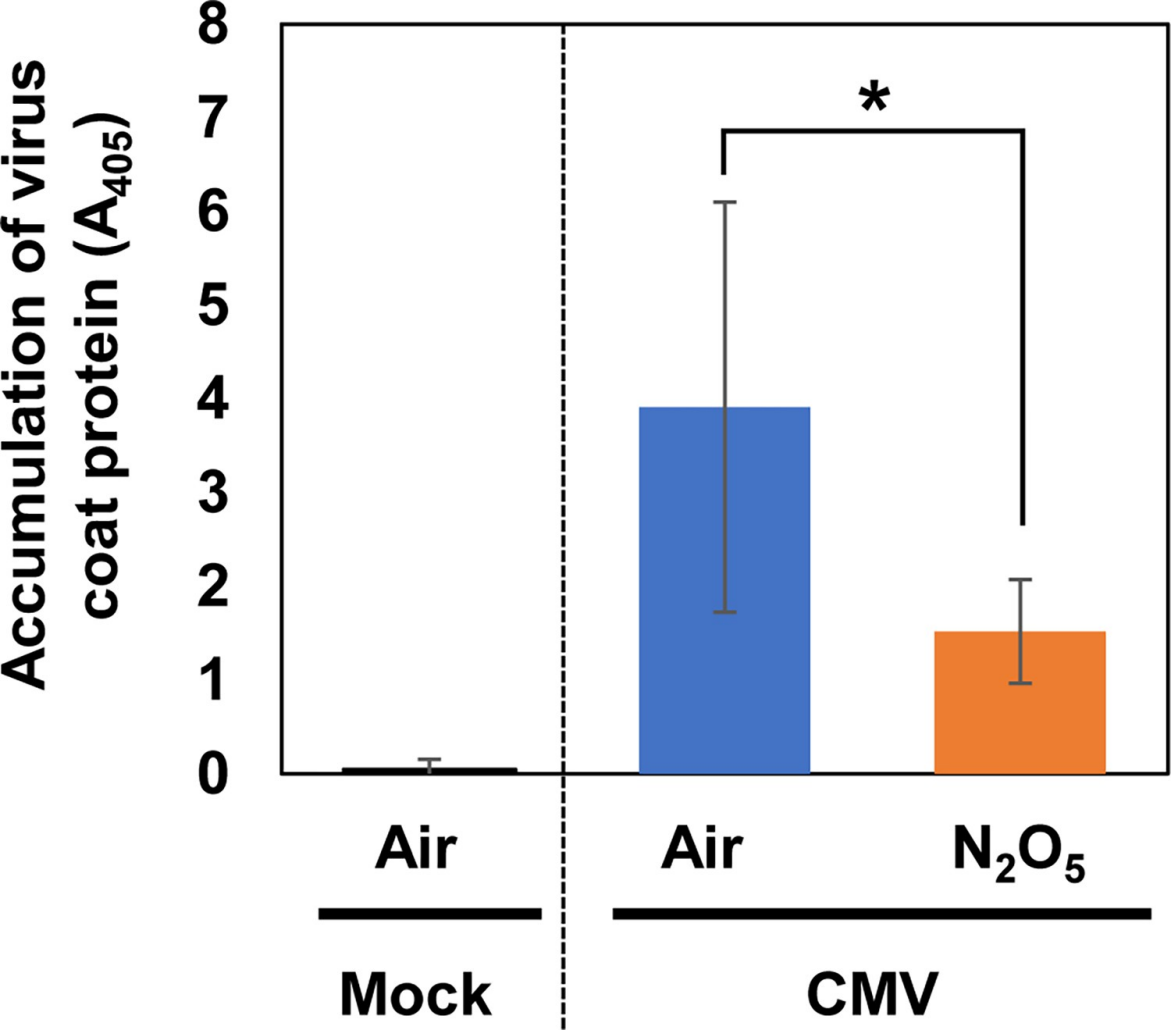
Induction of cucumber mosaic virus resistance in *Arabidopsis* plants by N_2_O_5_ gas exposure. *Arabidopsis* plants exposed to N_2_O_5_ gas were inoculated with CMV(Y). Two days after inoculation, the inoculated leaves were harvested and subjected to enzyme-linked immunosorbent assay using an antibody against the CMV CP. Asterisks denote significant differences (Student’s *t*-test, n = 6, *P* < 0.05).

Analysis of CMV resistance in the N_2_O_5_-gas-exposed plants was carried out. Two days after CMV inoculation, virus propagation in inoculated leaves was quantified by ELISA using anti-CMV CP antibody. The accumulation of CMV CP was significantly reduced in the N_2_O_5_-gas-exposed plants compared with air-exposed plants (Fig 4), which suggests that N_2_O_5_ gas exposure enhances CMV resistance.

### Changes in gene expression after exposure to N_2_O_5_ gas

To evaluate changes in global gene expression caused by exposure to N_2_O_5_ gas, RNA-Seq analysis was performed using N_2_O_5_-gas-exposed and air-exposed leaf samples at 24 h following the third exposure to N_2_O_5_ gas. The transcripts of 828 genes had increased in abundance by more than twofold after exposure to N_2_O_5_ gas (S2 Table), and the transcript abundances of 871 genes had decreased by less than half (S3 Table). The expression of several selected genes was checked by qRT-PCR and similar results were obtained (S2 Fig). Gene ontology term analysis of the genes exhibiting increased transcript abundance suggested that JA- and ET-dependent signaling pathways and disease resistance including systemic acquired resistance are activated by exposure to N_2_O_5_ gas (S3A Fig). Although GO term analysis showed that responses to SA and abscisic acid were suppressed by exposure to N_2_O_5_ gas (S3B Fig), the expression of *PR1*, a marker gene for responses involving SA, was induced (S2 Table).

**Fig 5:**
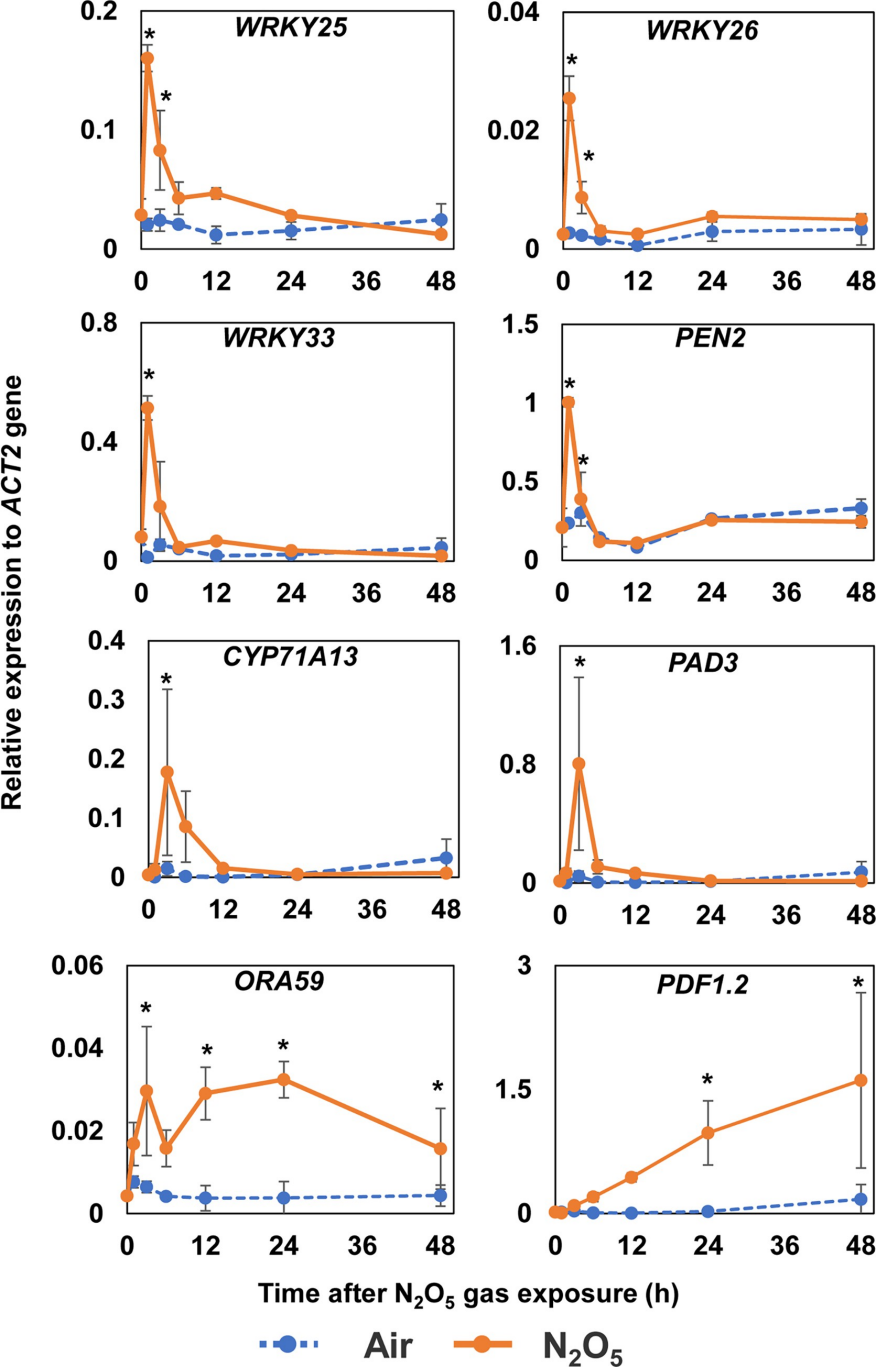
Changes in the expression of genes related to plant disease defense responses over time after exposure of *Arabidopsis* plants to N_2_O_5_ gas. The mRNA transcript abundances of genes related to plant disease defense, including *WRKY25*, *WRKY26*, *WRKY33*, *PEN2*, *CYP71A13*, *PAD3*, *ORA59*, and *PDF1*.*2*, were analyzed using qRT-PCR. Asterisks denote significant differences to 0 h samples (without N_2_O_5_ gas exposure; Dunnett’s test, n = 3, *P* < 0.05). *ACT2*, *ACTIN2*.

Among the genes exhibiting increased transcript abundance, we chose to further analyze changes in expression over time after N_2_O_5_ gas exposure of the defense-related genes *PAD3* and *PDF1*.*2* and transcription factors (TFs) *WRKY26* and *ORA59*, which may be involved in JA and ET responses. *Arabidopsis* plants were exposed to air (control) or N_2_O_5_ gas once for 20 s, and shoots of each individual plant were collected as independent samples for qRT-PCR at 1, 3, 6, 12, 24, and 48 h after exposure. Expression of *PAD3* and *WRKY26* transcripts was transiently induced within 3 h after N_2_O_5_ gas exposure (Fig 5). Protein encoded by *PAD3* gene is a cytochrome P450 involved in the biosynthesis of the antimicrobial compound camalexin from tryptophan (S6A Fig). Similarly, expression of transcripts of *CYP71A12*, *CYP71A13*, *PEN2*, and *NIT2*, which are involved in tryptophan metabolism (S6A Fig), was also induced (Fig 5 and S4 Fig), suggesting that tryptophan metabolism, including synthesis of camalexin and indole-glucosinolate derivatives, is enhanced by exposure of plants N_2_O_5_ gas. It has also been reported that the functions of *WRKY26* are redundant with those of the homologous *WRKY25* and *WRKY33* [32]. We also confirmed that the gene expression of *WRKY25* and *WRKY33* showed similar changes in gene expression as *WRKY26* after N_2_O_5_ gas exposure (Fig 5). These TFs might coordinate regulation of the response to treatment of plants with N_2_O_5_ gas. Meanwhile, *ORA59* showed biphasic induction of expression at around 3 h and 24 h after N_2_O_5_ gas exposure (Fig 5). The expression of *PDF1*.*2*, which encodes an antimicrobial peptide, gradually increased from 3 h after N_2_O_5_ gas exposure and remained at a high level until at least 48 h later (Fig 5). In contrast, the expression of *VSP2*, a gene specifically inducible by JA, was slightly responsive to N_2_O_5_ gas exposure with a significant but low level of induction at 12 h after N_2_O_5_ gas exposure (S4 Fig). In addition, although the expression of *PR1* was induced at 12 to 24 h after N_2_O_5_ gas exposure, it decreased to its basal level after 48 h (S4 Fig).

### Responses to N_2_O_5_ gas in *Arabidopsis* mutants deficient in phytohormone signaling pathways

**Fig 6:**
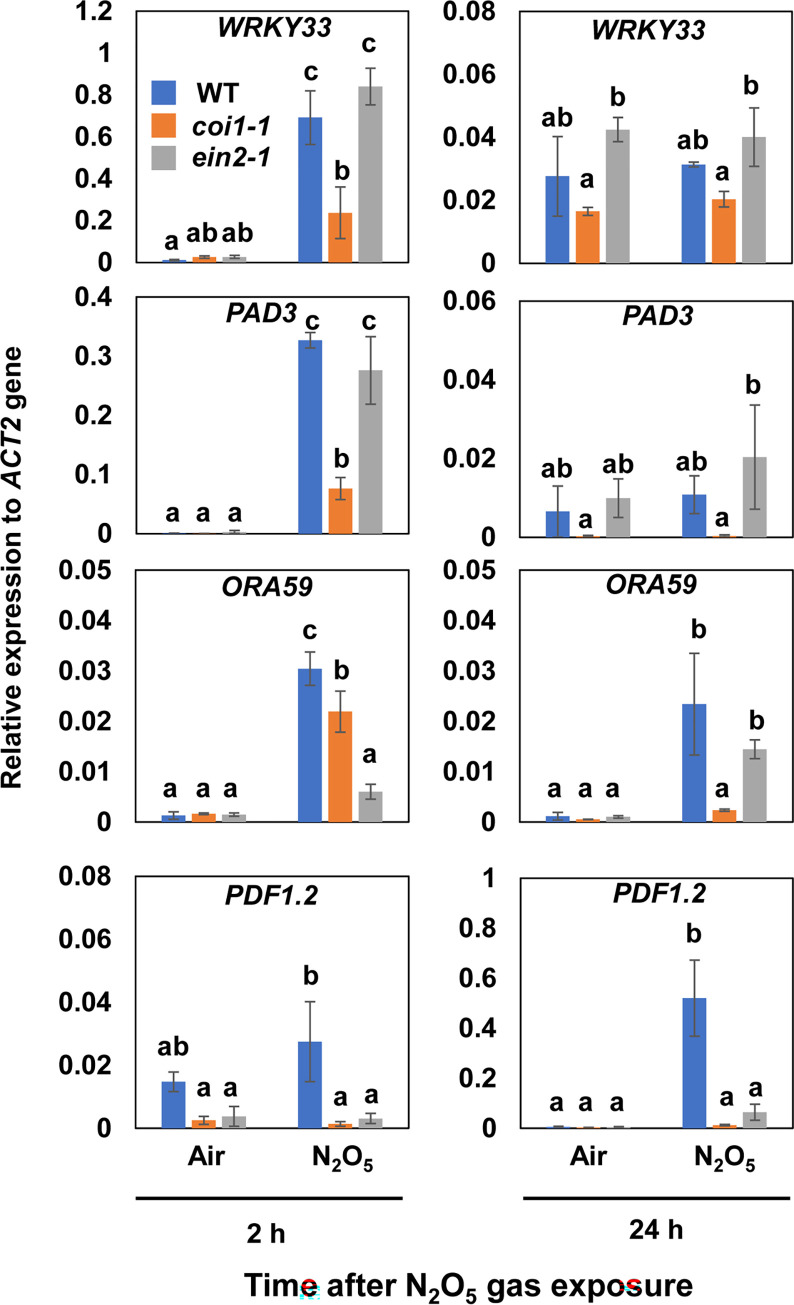
Expression of defense-related genes in *coi1-1* and *ein2-1* mutant *Arabidopsis* plants after exposure to N_2_O_5_ gas. The accumulation of mRNA transcripts of defense-related genes, including *WRKY33*, *PAD3*, *ORA59*, and *PDF1*.*2* was analyzed in wild-type, *coi1-1*, and *ein2-1 Arabidopsis* plants. Different letters denote significant differences among treatments (Tukey–Kramer test, n = 3, *P* < 0.05). *ACT2*, *ACTIN2*.

To determine whether the observed responses to N_2_O_5_ gas exposure are mediated by the JA and ET signaling pathways, we exposed mutant plants for each of these phytohormone signaling pathways to N_2_O_5_ gas and analyzed their responses. *Arabidopsis* plants were exposed to air (control) or N_2_O_5_ gas for 20 s, and shoots of each individual plant were harvested at 2 and 24 h later for qRT-PCR The induction of *WRKY33*, *WRKY26*, *PEN2*, and *PAD3* expression was reduced in *coi1-1*, a JA signaling mutant at 2 h after N_2_O_5_ gas exposure, whereas expression of these genes was similar to the wild type in *ein2-1*, an ET signaling mutant (Fig 6 and S5 Fig). The transcript abundance of *ORA59* was relatively lower in plants carrying either mutation especially *ein2-1*, at 2 h after N_2_O_5_ gas exposure. The transcript abundance of *ORA59* was significantly lower only in *coi1-1* at 24 h after exposure to N_2_O_5_ gas. The contribution of ET and JA to the regulation of *ORA59* expression seems to differ between 2 h and 24 h after N_2_O_5_ gas exposure (Fig 6). Induction of the expression of *PDF1*.*2* was greatly attenuated in both the *coi1-1* and *ein2-1* mutants, especially at 24 h after N_2_O_5_ gas exposure (Fig 6). These results indicated that both JA and ET signaling have important roles in the activation of gene expression by N_2_O_5_ gas.

**Fig 7:**
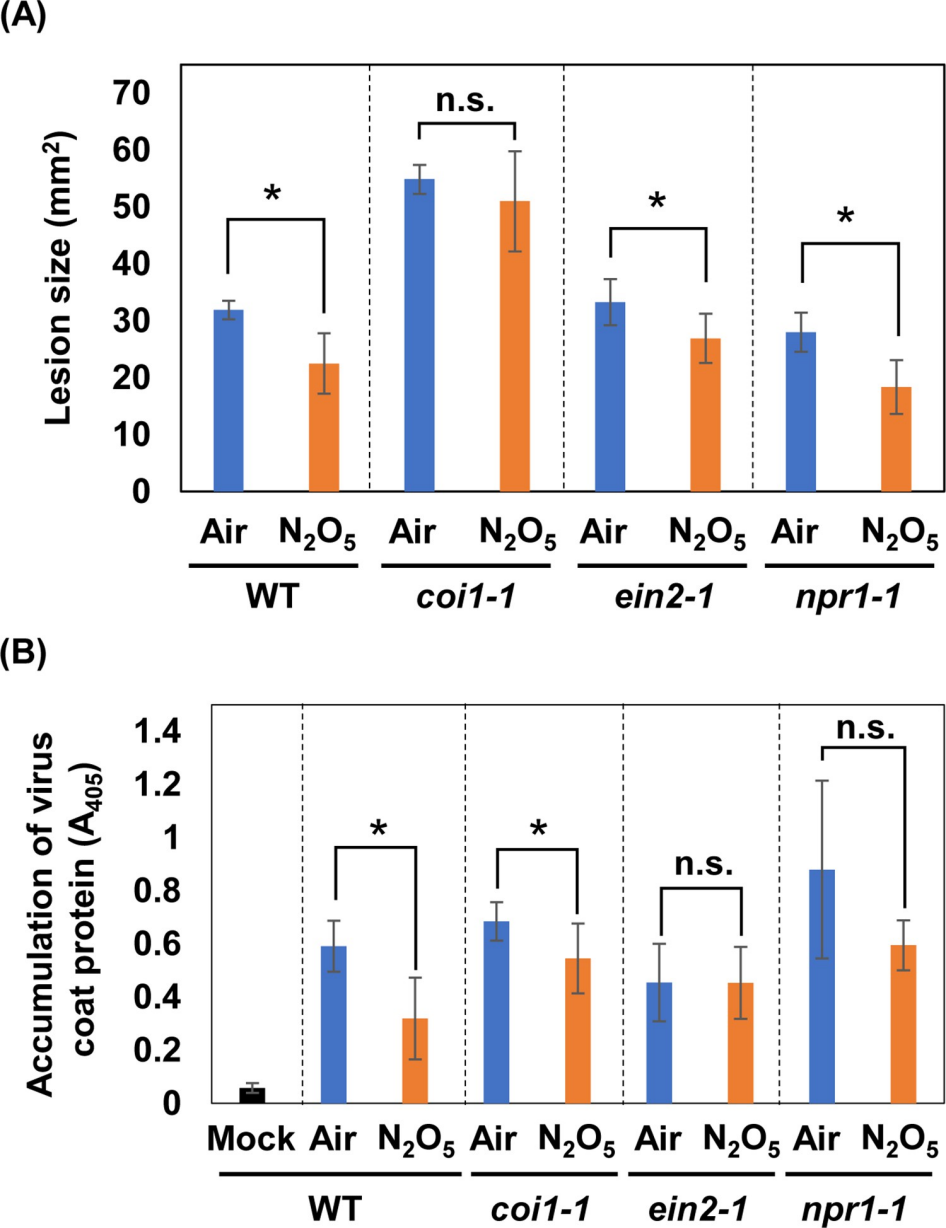
Resistance induced by N_2_O_5_-gas against *Botrytis cinerea* and CMV in *Arabidopsis* phytohormone signaling mutants. (A) The areas of the lesions at 2 days after *B*. *cinerea* inoculation were measured and the mean (± standard deviation) is shown. Asterisks denote significant differences (Student’s *t*-test, n = 9, *P* < 0.05). (B) Two days after CMV(Y) inoculation, the inoculated leaves were harvested and subjected to ELISA. Asterisks denote significant differences (Student’s *t*-test, n = 6, *P* < 0.05). n.s.: not significant.

In addition, we analyzed the induction of disease resistance by exposure to N_2_O_5_ gas in *coi1-1* and *ein2-1* mutants. The SA signaling mutant *npr1-1* was also used in these experiments. Wild-type, *coi1-1*, *ein2-1*, and *npr1-1* plants were exposed to N_2_O_5_ gas under the same conditions as in Fig 2. The sizes of lesions caused by *B*. *cinerea* infection were reduced with exposure to N_2_O_5_ gas as compared with the Air-control in *ein2-1* and *npr1-1* but were not significantly different in *coi1-1* as shown in Fig 7A. These results indicated that JA signaling has a major role in the enhancement of *B*. *cinerea* resistance by N_2_O_5_ gas. In contrast, the induction of CMV resistance by exposure to N_2_O_5_ gas was compromised in *ein2-1* and *npr1-1*, but was maintained in *coi1-1* (Fig 7B). In particular, the effect of the N_2_O_5_ gas tended to be weak in *ein2-1*, suggesting that ET may be a major factor in CMV resistance induced by N_2_O_5_ gas.

## Discussion

In this study, we showed that exposure of *Arabidopsis thaliana* to N_2_O_5_ gas produced from air-derived plasma can activate plant immunity mainly through JA and ET signaling. Specifically, we showed that exposure of plants to N_2_O_5_ gas enhanced *B*. *cinerea* and CMV resistance but not *Pst* resistance. However, it is important to note that although N_2_O_5_ gas is composed mainly of highly concentrated N_2_O_5_, it also contains a certain amount of the other active species such as O_3_ and NO_2_ (S1C Fig). Methods for exogenously exposing plants to ROS or RNS in a gaseous state for disease control have been reported using O_3_ and NO_2_ [18, 33]. Increased production of SA, ET, and JA has been reported upon O_3_ exposure in *A*. *thaliana* [34–36]. Ethylene is thought to be involved in O_3_ sensitivity because O_3_-induced damage is reduced in ethylene-deficient mutants. However, ET production is suppressed and damage is reduced in MeJA-treated plants after O_3_ exposure, suggesting that JA acts antagonistically with ET [34]. With regard to SA, the accumulation of SA after O_3_ exposure was high in the ET-overproducing mutant *eto1*, whereas ET production was low in the SA-deficient mutant, suggesting that ET and SA work cooperatively after O_3_ exposure [35]. Ozone exposure also effectively inhibits the propagation of several plant viruses such as tobacco mosaic virus and soybean mosaic virus [37, 38]. In the present study, the N_2_O_5_ gas exposure shown to enhance CMV resistance was dependent mainly on ET signaling pathways according to our analysis using phytohormone signaling mutants (Fig 7B). Ethylene signaling is also partially involved in the CMV resistance conferred by RCY1, a disease-resistance protein in *A*. *thaliana* [39]. The CMV resistance induced by N_2_O_5_ gas and that conferred by RCY1 might employ a common ET-mediated mechanism. Meanwhile, although activation of SA responses was not detected during the GO term analysis of our RNA-Seq results, transient activation of *PR1* expression was confirmed (S3B and S4 Figs and S2 Table). These results imply that SA signaling may also be partially activated. The fact that there was no significant enhancement of CMV resistance by N_2_O_5_ gas exposure in the *npr1-1* mutant supports this possibility (Fig 7B). Because a cooperative function of ET and SA has also been reported for O_3_ [35], it is possible that O_3_ contained in the N_2_O_5_ gas partially contributes to the CMV resistance induced by the N_2_O_5_ gas. Otherwise, N_2_O_5_ has a strong oxidative effect much like that of O_3_, so an unknown mechanism might be operating due to an oxidative effect of N_2_O_5_. Further analysis is needed to understand the mechanistic details of the enhancement of CMV resistance by N_2_O_5_ gas.

*Botrytis cinerea* resistance is regulated by a complex network of SA, ET, and JA signals [40]. However, JA and ET play major roles in resistance to necrotrophic pathogens including *B*. *cinerea* [41]. An increase in SA content has been observed immediately after exposure in the case of exposure to NO_2_ gas [18]. Interestingly, gene expression related to the synthesis and metabolism of JA is also activated after exposure to NO_2_ gas. Also, a reduction in the amount of active JA and accumulation of its metabolites is observed [18]. Exposure to NO_2_ gas enhances *B*. *cinerea* resistance, but this effect is impaired in both SA-deficient *NahG* plants and JA biosynthetic mutants, suggesting that NO_2_-induced resistance enhancement involves not only SA accumulation but also activation of JA metabolism [18]. In contrast, our results suggest that N_2_O_5_ gas enhances resistance to *B*. *cinerea* mainly by activating JA signaling (Figs 2 and 7A). Gene expression analysis suggests that JA and ET signals are activated in a coordinated manner by exposure to N_2_O_5_ gas (S3A Fig). Analysis using phytohormone signaling mutants also supports that JA and ET signals are activated by exposure to N_2_O_5_ gas (Fig 6). However, GO term analysis of our RNA-Seq results suggests that SA signaling was either not activated or was somewhat suppressed by the exposure of plants to N_2_O_5_ gas (S3 Fig), although transient induction of *PR1* gene was observed (S4 Fig). These findings suggest that different regulatory mechanisms likely operate in the *B*. *cinerea* resistance induced by NO_2_ gas or N_2_O_5_ gas.

In general, SA plays a central role in resistance to *Pst*, while JA and ET act antagonistically [42, 43]. However, JA is reported to play a cooperative role with SA in the induction of *Pst* resistance by oligogalacturonides and chitosan oligosaccharides [44, 45]. In the present study, we did not observe any enhancement of *Pst* resistance by the N_2_O_5_ gas (Fig 3), which might have been because SA signaling was not predominantly activated compared with JA (S3 Fig). Because NO_2_-gas exposure enhances *Pst* resistance via SA signaling [18], the differential effects of N_2_O_5_ gas and NO_2_ gas on disease resistance can be confirmed again.

A key transcription factor, WRKY33, controls the expression of genes regulating resistance to *B*. *cinerea* via ET/JA signaling and camalexin biosynthesis such as *CYP71A12*, *CYP71A13*, and *PAD3* [46, 47]. The transient increase in the abundance of *WRKY33* transcripts by exposure N_2_O_5_ gas suggests activation of WRKY33-mediated *B*. *cinerea* resistance (Fig 5). Because the N_2_O_5_ gas induced the expression of *CYP71A12*, *CYP71A13*, *PAD3*, and *PEN2* (Fig 5), it is likely that secondary metabolites derived from tryptophan such as camalexin, indole-glucosinolate derivatives, and indole-carboxylic acid (S6A Fig) are involved in the N_2_O_5_-gas-induced *B*. *cinerea* resistance. Furthermore, *WRKY25* and *WRKY26*, which are functionally redundant with *WRKY33* [32], are thought to regulate these metabolic systems in a coordinated manner (Fig 5). However, it has been reported that the induction of *B*. *cinerea* resistance by NO_2_ is *PAD3*-dependent but not accompanied by an increase in camalexin content [18]. Further analysis is needed to determine which metabolites, including camalexin, contribute to the enhancement of *B*. *cinerea* resistance by N_2_O_5_ gas. Meanwhile, although exposure to N_2_O_5_ gas strongly induced the expression of *ORA59* and *PDF1*.*2* (Fig 5), the induction of *VSP2* was relatively weak (S4 Fig). The expression of *ORA59*, which encodes a TF, is regulated by both ET and JA and controls the expression of *PDF1*.*2*, which is involved in disease resistance (S6B Fig). However, *VSP2* is thought to function in wounding response and insect resistance under the control of the TF MYC2 in a JA-specific manner (S6B Fig) [14, 48]. It is possible that ET signaling becomes more dominant in the relationship between ORA59 and MYC2 during exposure to N_2_O_5_ gas.

Interestingly, the response of *WRKY33* transcript to exposure to N_2_O_5_ gas is highly similar to the responses to treatments with damage-associated molecular patterns (DAMPs) such as HMGB3 and Pep1 [49]. In plants and animals, DAMPs released from cells due to injury induce immune responses [50, 51]. Therefore, exposure to N_2_O_5_ gas causes slight cellular damage (Fig 1), which might result in the release of DAMPs into the apoplast of plant tissues. Proteinaceous DAMPs such as HMGB1 and HSPs are known to play an important role in the inflammatory response in animal cells [52]. Pattern recognition receptors (PRR, *e*.*g*., Toll-like receptor 4) recognize DAMPs released from damaged cells, and thereby transmit the damage stimulus to surrounding cells [52]. Furthermore, post-translational modifications of DAMPs are known to alter their functions [53]. A recent report indicates that proteinaceous DAMPs, in which tyrosine residues have been modified by nitration, activate the PRR more strongly than do unmodified DAMPs in HeLa cells [54]. Dinitrogen pentoxide is a powerful oxidizing and nitrating agent, and is an important agent widely used in the nitration and *S*-nitrosylation of organic compounds, as for the production of nitrotyrosine when tyrosine is treated with the N_2_O_5_ gas [6]. Therefore, exposure to the N_2_O_5_ gas might contribute not only to the release of DAMPs, but also to the modification of DAMPs by nitration or *S*-nitrosylation to influence their function in plant immunity. The involvement of DAMPs in the enhancement of disease resistance by N_2_O_5_ gas will be elucidated in future analyses.

In conclusion, we have shown that N_2_O_5_ gas has potential for development as a new technology for plant disease control. N_2_O_5_ is converted to nitric acid by reacting with water, and it can be used by plants as a source of nitrogen. Therefore, treatment with N_2_O_5_ gas would be almost free from risks of environmental pollution. In addition because the amount of electricity required for production of N_2_O_5_ gas is relatively low [6], control of plant diseases using N_2_O_5_ gas could contribute as a low-cost and environmentally friendly technology to the establishment of a sustainable agricultural system. Furthermore, the device used in this study can selectively supply O_3_ or NO/NO_2_ by mode switching [6]. Since the present study was performed under laboratory conditions using *A*. *thaliana*, validation under field conditions using crops is a subject for future work. However, this type of approach might be useful for efficiently controlling plant diseases by exposing crop species to the appropriate active gas composition for the type of disease. Recently, Kumar and co-workers reported that glycine betaine and *Arbuscular mycorrhizal* fungi treatment reduces chromium toxicity via reduction of oxidative stress [55–57]. Combining such treatments with N_2_O_5_ gas exposure may allow for the development of more effective and harmless disease control methods.

## Supporting information

S1 Fig Atmospheric-pressure plasma device for the generation of N_2_O_5_ gas. The atmospheric-pressure plasma device was developed at the Graduate School of Engineering, Tohoku University [6]. (A) Device installation status. (B) Photographs showing exposure of *Arabidopsis thaliana* plants to N_2_O_5_ gas. The area in the box in (A). (C) Typical densities of reactive species in the gas generated by the plasma device.
(PDF)

S2 Fig Analysis of N_2_O_5_-gas-inducible gene expression in *Arabidopsis thaliana* plants by qRT-PCR. To confirm the results of RNA-Seq, five genes with elevated transcript expression after exposure to N_2_O_5_ gas were chosen for further analysis of their relative transcript abundances by qRT-PCR. *Arabidopsis* plants were exposed to air (control) or N_2_O_5_ gas for 20 s once a day for 3 days. Shoots of each individual plant were collected as independent samples at 24 h after the third exposure. Total RNA was extracted and subjected to analysis of the relative mRNA transcript abundances of defense-related genes, including *PDF1*.*2*, *PDF1*.*4*, *ORA59*, *WRKY26*, and *PR4*. Data were normalized to *ACTIN2* mRNA transcript abundance. Asterisks denote significant differences relative to the air control (Student’s *t*-test, n = 3, *P* < 0.05). *ACT2*, *ACTIN2*. The fold change (N_2_O_5_/Air) calculated from qRT-PCR was compared to the fold change obtained from RNA-seq.
(PDF)

S3 Fig Gene Ontology term analysis of genes with increased or decreased transcript abundance after exposure to N_2_O_5_ gas. Genes with increased or decreased transcript abundance after exposure to N_2_O_5_ gas from the RNA-Seq results were subjected to Gene Ontology term analysis. Enrichment of functional categories was defined implementing Gene Ontology tool online (http://geneontology.org/). Fold enrichment of genes exhibiting increased (A) or decreased (B) transcript abundance after exposure to N_2_O_5_ gas is shown.
(PDF)

S4 Fig Changes in the expression of genes related to plant disease defense responses over time after exposure to N_2_O_5_ gas. The gene expression of *CYP71A12*, *NIT2*, *VSP2*, and *PR1* was analyzed by qRT-PCR as in Fig 5.
(PDF)

S5 Fig Expression of plant disease defense-related genes in *coi1-1* and *ein2-1* mutants after exposure to the N_2_O_5_ gas. The gene expression of *WRKY26* and *PEN2* was analyzed by qRT-PCR as in Fig 6.
(PDF)

S6 Fig Model of tryptophan metabolism pathway and crosstalk between JA and ET signaling in *Arabidopsis thaliana*. (A) Model of tryptophan metabolism pathway. 4MI3G, 4-methoxy indolyl-3-methyl glucosinolate. (B) Model of JA and ET signaling crosstalk. Arrows indicate positive effects. Negative interaction of ORA59 and MYC2 is known. The genes analyzed in this study are shown in red letters.
(PDF)

S1 Table Primers used in this study. (XLSX)

S2 Table Genes whose transcript abundance increased more than twofold after exposure to N_2_O_5_ gas compared to air control in RNA-seq analysis. (XLSX)

S3 Table Genes whose transcript abundance decreased less than half after exposure to N_2_O_5_ gas compared to air control in RNA-seq analysis. (XLSX)
