# Supplementary figures and images for: Activation of plant immunity by exposure to dinitrogen pentoxide gas generated from air using plasma technology
Source: PLoS One. 2022 Jun 24;17(6):e0269863. doi: 10.1371/journal.pone.0269863 (PMC9231731; doi:10.1371/journal.pone.0269863)

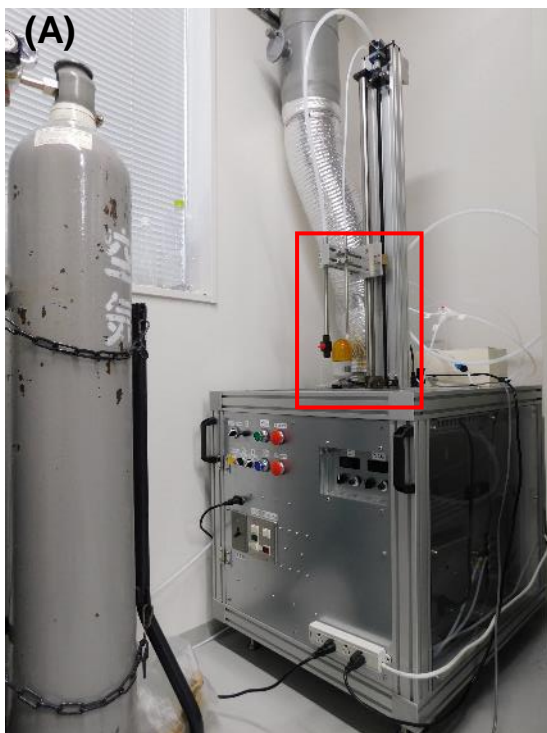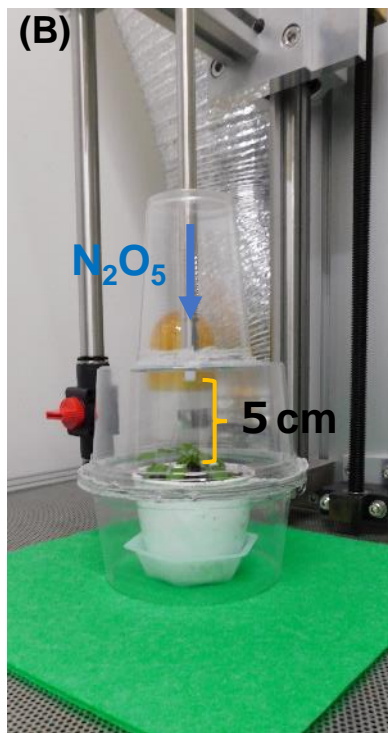

(C)

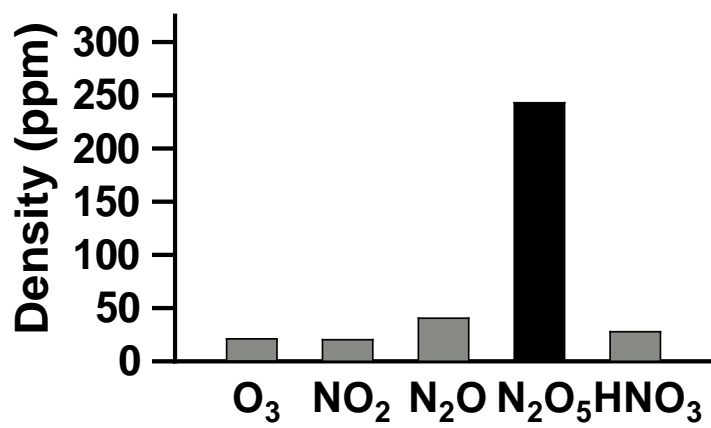

Supplement: S1 Fig — The atmospheric-pressure plasma device was developed at the Graduate School of Engineering, Tohoku University [6]. (A) Device installation status. (B) Photographs showing exposure of Arabidopsis thaliana plants to N2O5 gas. The area in the box in (A). (C) Typical densities of reactive species in the gas generated by the plasma device. (PDF) [file pone.0269863.s001.pdf]

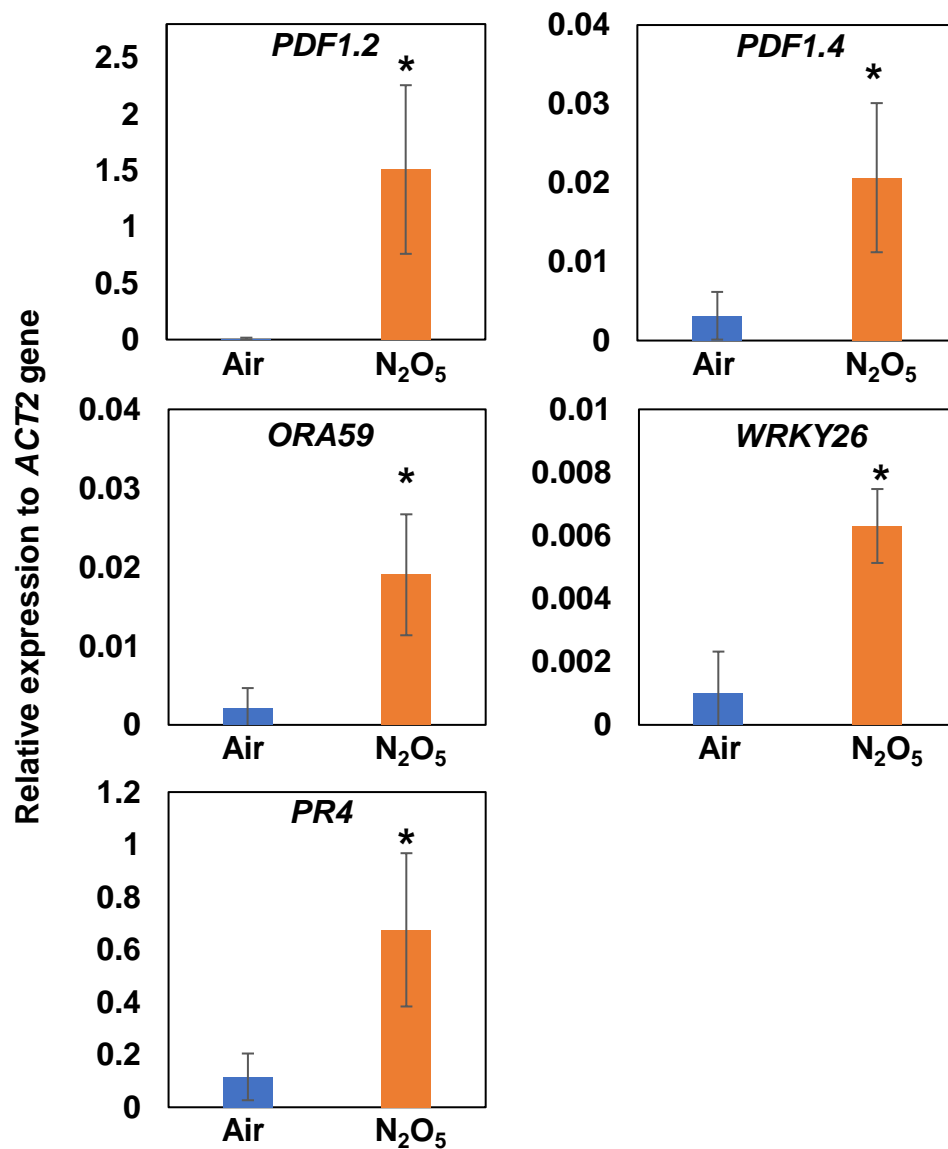

|               | Fold change ( $N_2O_5$ /Air) |         |
|---------------|------------------------------|---------|
|               | RNA-seq                      | qRT-PCR |
| <i>PDF1.2</i> | 139                          | 217     |
| <i>PDF1.4</i> | 7.07                         | 6.56    |
| <i>ORA59</i>  | 24.6                         | 8.84    |
| <i>WRKY26</i> | 13.1                         | 6.36    |
| <i>PR4</i>    | 5.53                         | 5.83    |

Supplement: S2 Fig — To confirm the results of RNA-Seq, five genes with elevated transcript expression after exposure to N2O5 gas were chosen for further analysis of their relative transcript abundances by qRT-PCR. Arabidopsis plants were exposed to air (control) or N2O5 gas for 20 s once a day for 3 days. Shoots of each individual plant were collected as independent samples at 24 h after the third exposure. Total RNA was extracted and subjected to analysis of the relative mRNA transcript abundances of defense-related genes, including PDF1.2, PDF1.4, ORA59, WRKY26, and PR4. Data were normalized to ACTIN2 mRNA transcript abundance. Asterisks denote significant differences relative to the air control (Student’s t-test, n = 3, P < 0.05). ACT2, ACTIN2. The fold change (N2O5/Air) calculated from qRT-PCR was compared to the fold change obtained from RNA-seq. (PDF) [file pone.0269863.s002.pdf]

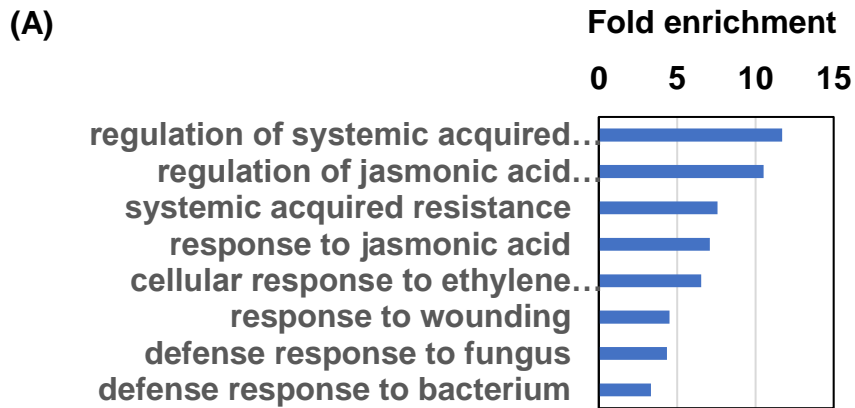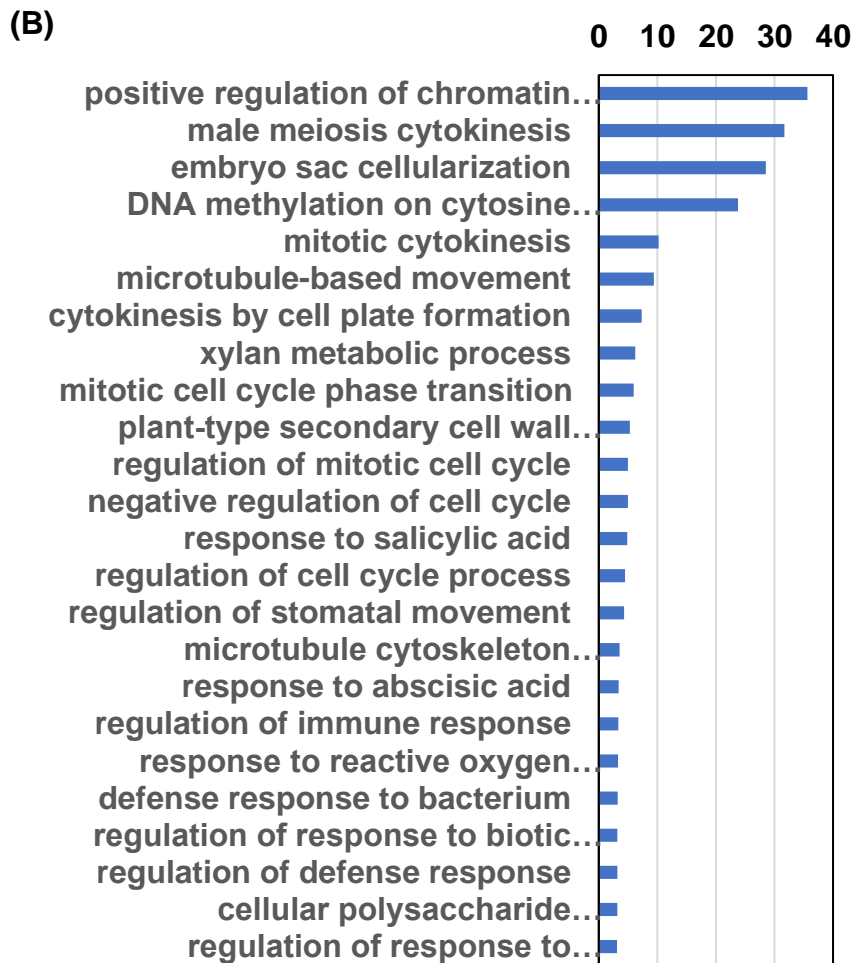

Supplement: S3 Fig — Genes with increased or decreased transcript abundance after exposure to N2O5 gas from the RNA-Seq results were subjected to Gene Ontology term analysis. Enrichment of functional categories was defined implementing Gene Ontology tool online (http://geneontology.org/). Fold enrichment of genes exhibiting increased (A) or decreased (B) transcript abundance after exposure to N2O5 gas is shown. (PDF) [file pone.0269863.s003.pdf]

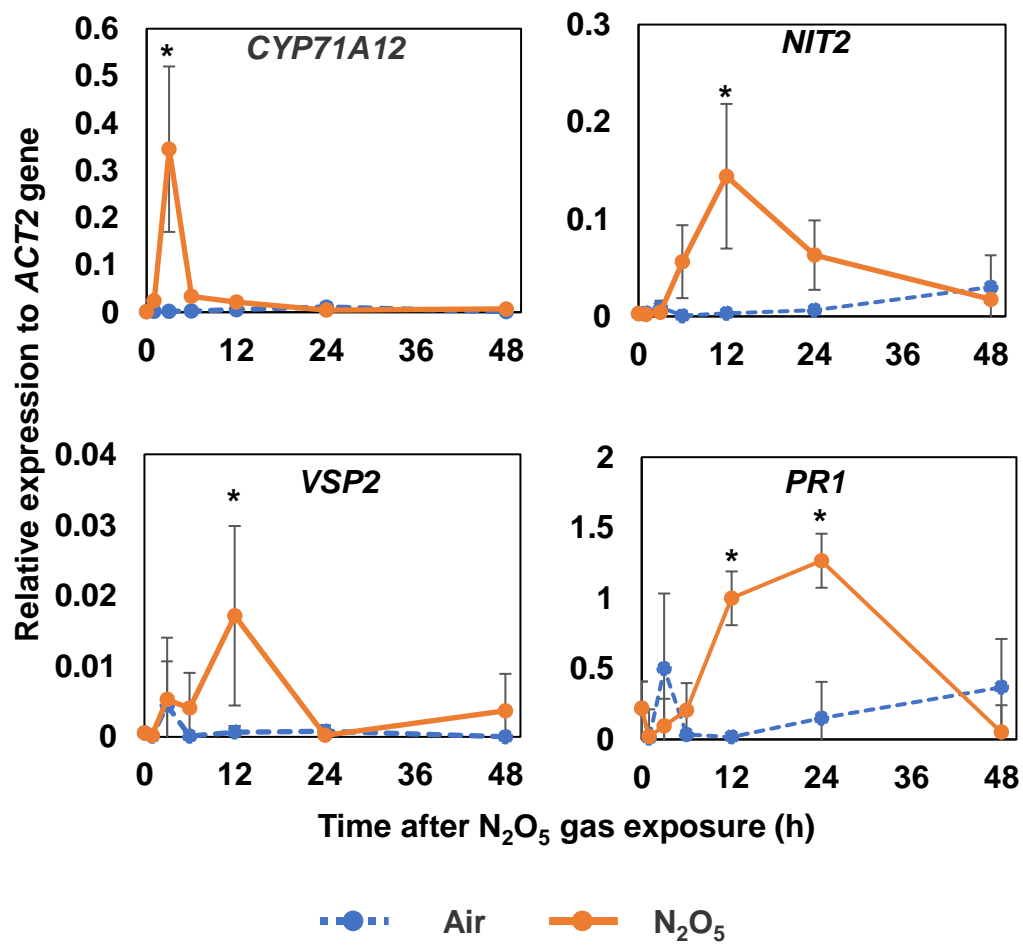

Supplement: S4 Fig — The gene expression of CYP71A12, NIT2, VSP2, and PR1 was analyzed by qRT-PCR as in Fig 5. (PDF) [file pone.0269863.s004.pdf]

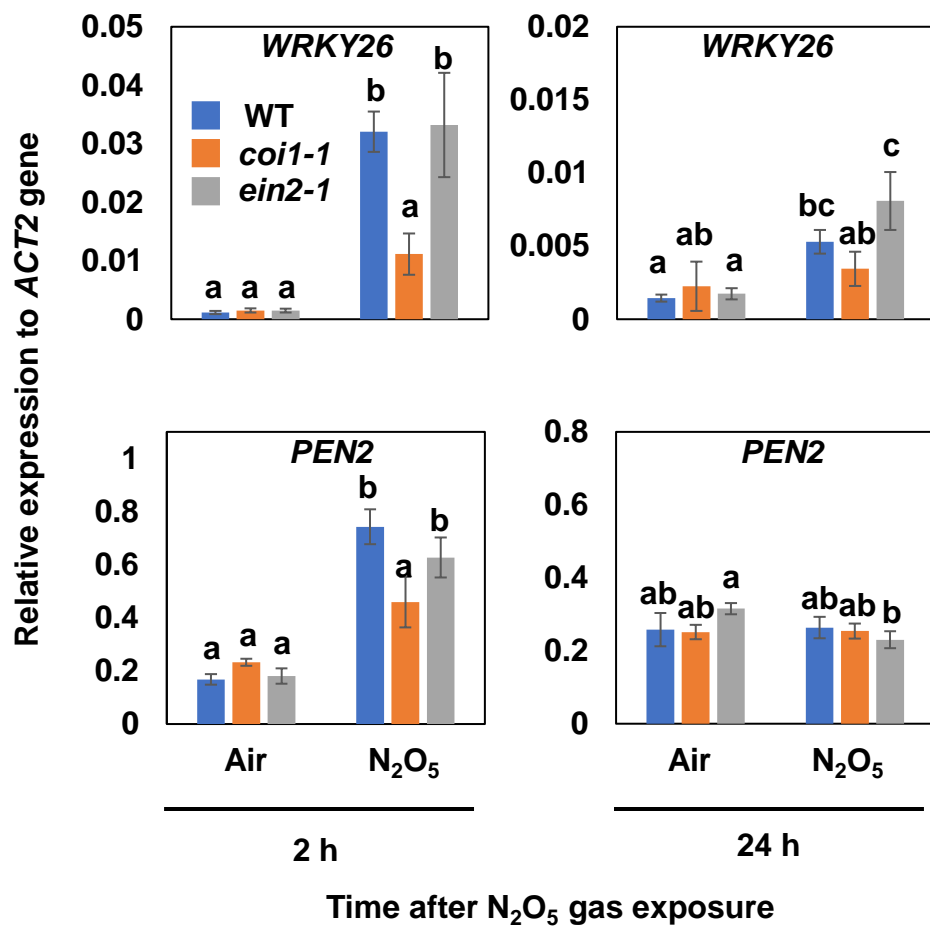

Supplement: S5 Fig — The gene expression of WRKY26 and PEN2 was analyzed by qRT-PCR as in Fig 6. (PDF) [file pone.0269863.s005.pdf]

(A)

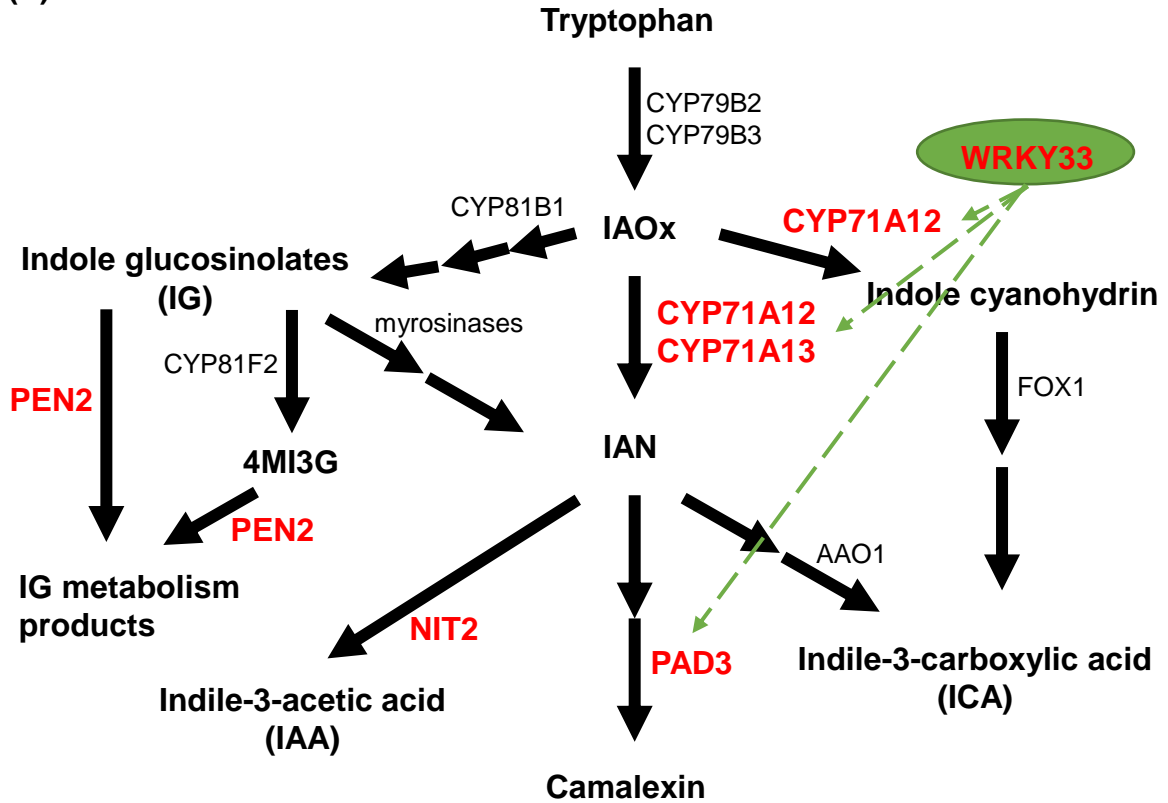

(B)

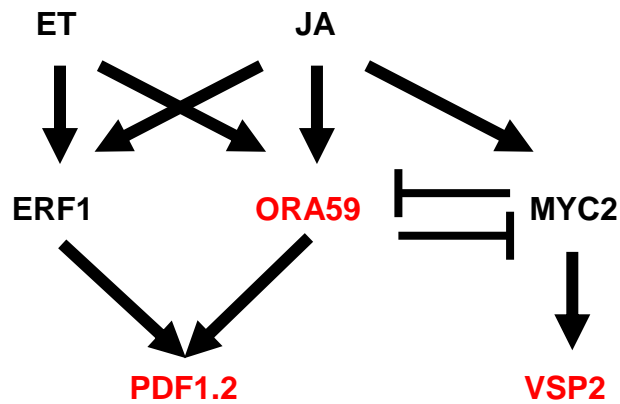

Supplement: S6 Fig — (A) Model of tryptophan metabolism pathway. 4MI3G, 4-methoxy indolyl-3-methyl glucosinolate. (B) Model of JA and ET signaling crosstalk. Arrows indicate positive effects. Negative interaction of ORA59 and MYC2 is known. The genes analyzed in this study are shown in red letters. (PDF) [file pone.0269863.s006.pdf]
